# A Rare Case of Ganser Syndrome: Psychogenic or Organic?

**DOI:** 10.7759/cureus.10144

**Published:** 2020-08-30

**Authors:** Eduardo D Espiridion, Kyra Valent, Anas Qatanani, Oluwakemi Adesina, Adeolu O Oladunjoye

**Affiliations:** 1 Psychiatry, Drexel University College of Medicine, Philadelphia, USA; 2 Psychiatry, West Virginia School of Osteopathic Medicine, Lewisburg, USA; 3 Psychiatry, West Virginia University School of Medicine, Martinsburg, USA; 4 Psychiatry, Philadelphia College of Osteopathic Medicine, Philadelphia, USA; 5 Psychiatry, Reading Hospital - Tower Health, West Reading, USA; 6 Medicine, Philadelphia College of Osteopathic Medicine, Philadelphia, USA; 7 Medicine, Drexel University College of Medicine, Philadelphia, USA; 8 Medical Critical Care, Boston Children's Hospital, Boston, USA

**Keywords:** ganser syndrome, psychogenic, organic, dissociative disorder, approximate answer

## Abstract

Ganser syndrome (GS) is a rare neurological disorder characterized by answer approximation, clouded consciousness, somatic conversion symptoms, and visual or auditory hallucinations. The objective of this case report is to elucidate the presentation of a patient with GS and to highlight the interplay of psychological and organic determinants in this condition.

We present a 66-year-old man with a history of concussion and short-term memory loss who presented with selective, remote, and recent memory loss following the death of his wife, visual hallucinations, approximation of answers regarding his current state, and limited insight into his condition. We found the patient oriented only to place and person, with impaired short-term memory and no language abnormalities. Montreal cognitive assessment (MOCA) exam showed mild-to-moderate cognitive impairment. The patient's presentation can be explained by both psychological and organic causes. Negative results from imaging and testing showed that the patient's recent emotional stressor, the death of his wife, may be contributing to the current state. However, the patient also has a history of hospitalization for traumatic brain injury (TBI) and a recent history of progressive memory loss. Therefore, the combination of psychological and organic factors likely played supplementary roles in the patient's current presentation. This case supports the literature that GS is a psychogenic disorder. However, an organic cause from the long-term sequelae of TBI needs further exploration.

## Introduction

Ganser syndrome (GS) is a very rare condition first described in 1897 by Sigbert Ganser [1]. He described it as a transitory symptom of mental illness characterized by giving approximate answers when questioned, dulling of consciousness, hysterical neurological changes, and hallucinations [2]. He stated these symptoms were abrupt and complete in onset and remission. Later, in 1979, Enoch and Trethowan expanded on Ganser’s symptomatology and described GS based on four core clinical features: approximate answers, clouding of consciousness, somatic conversion symptoms, and optional visual or auditory pseudohallucinations [3]. However, not all clinical features are needed for making the diagnosis [4]. The most essential symptom of GS is answer approximation when asked basic questions, but this symptom is neither diagnostic nor pathognomonic for GS [1].

The etiology of GS is still not well understood. This has led to different controversies regarding whether it is associated with a purely psychiatric disorder (hysterical dissociative state) or an organic cause [5]. GS is primarily considered a psychological disorder in response to extreme stressors. Enoch et al. and Haddad also found GS in comorbid states of schizophrenia and affective disorders [4, 6-7]. However, there have been reports of an association with organic brain conditions such as traumatic brain injury (TBI), stroke, and other forms of brain injuries, mostly those involving the frontal lobes [1, 8]. The difficulty with making the diagnosis of GS stems from the common overlap with organic or other psychiatric disorders [9].

We present the case of a 66-year-old man whose presentation provides insight into the psychological and organic etiologies associated with GS, which may help clarify the connection between these two causes and its symptomatology. 

## Case presentation

Presenting complaints

A 66-year-old Caucasian male presented to the ED with memory loss, altered mental status, and agitation. He was brought to the ED by police after bystanders noticed him sitting in the car with his pets for two hours at a gas station. Initially, he did not recall that he had pets with him when he was picked up and claimed he was at a contest before arriving at the ED. Two days later, he was able to recall being in the car with his pets and claimed he was picked up by the police for leaving his animals unattended, but he had no recollection of how, when, and why he was at the gas station. The patient claims he lives with his wife, who died two days prior to presentation, and mother, who died a few years earlier. He says he just saw them leave the room while he was being seen in the hospital. He had no recollection of his wife’s death or the earlier phone call. The patient reports a remote history of hitting his head while playing soccer back in college, approximately 40 years ago, and was hospitalized for about a month. However, he could not remember whether he had any complications from the episode of head trauma.

He presents with selective remote and recent memory loss. He approximates answers to questions about the number of children he has, wives ever married in the past, and events that led to his present hospitalization. He cannot answer questions promptly. The patient drinks alcohol occasionally and has a history of occasional marijuana use during young adulthood. He smoked 1.5 packs/day of cigarettes for 17 years and stopped five years ago. Past medical history reveals a history of memory problems with an onset of 18 months ago. He states that his problem prevented him from being able to work the machines at his job, causing him to take a leave of absence. A few months before present admission, his primary care physician (PCP) assessed him for memory loss and treated him for early onset dementia with donepezil 5 mg twice daily, but he was not compliant with this medication. He has a known history of hypertension, hyperlipidemia, and degenerative disc disease and denies depression, anxiety, or any suicidal thoughts.

Examination

The patient appears unkempt. He is calm, cooperative, and listens to simple instructions. The patient’s speech is not fluent but has an appropriate tone. His mood is sad as he worries about his animals, and his affect is mood-congruent. He is conscious and alert, but only oriented to self and place. He is not oriented to time, often giving the incorrect month (September instead of July) and incorrect year (answer ranges from 1960s to 2060s instead of 2020). But he is aware of the current president. Language evaluation reveals no abnormalities, as the patient can name objects, repeat a sentence, and follow commands adequately. He has visual hallucinations about seeing his wife and mother, who are both deceased. He has no tangentially in thought. Montreal cognitive assessment (MOCA) score is 18/30, with 0/3 objects remembered. He was unable to spell "WORLD" backward. His remote memory is good as he describes his college life, past jobs, and other events in his past with details. His recent memory is poor as he is unsure how he got to the hospital and will give an approximate answer to why he is in the hospital. He has limited insight into his condition. He admits to having memory problems before this hospitalization; however, he is unaware of his approximation and error when answering questions. 

Investigation and treatment

**Figure 1:**
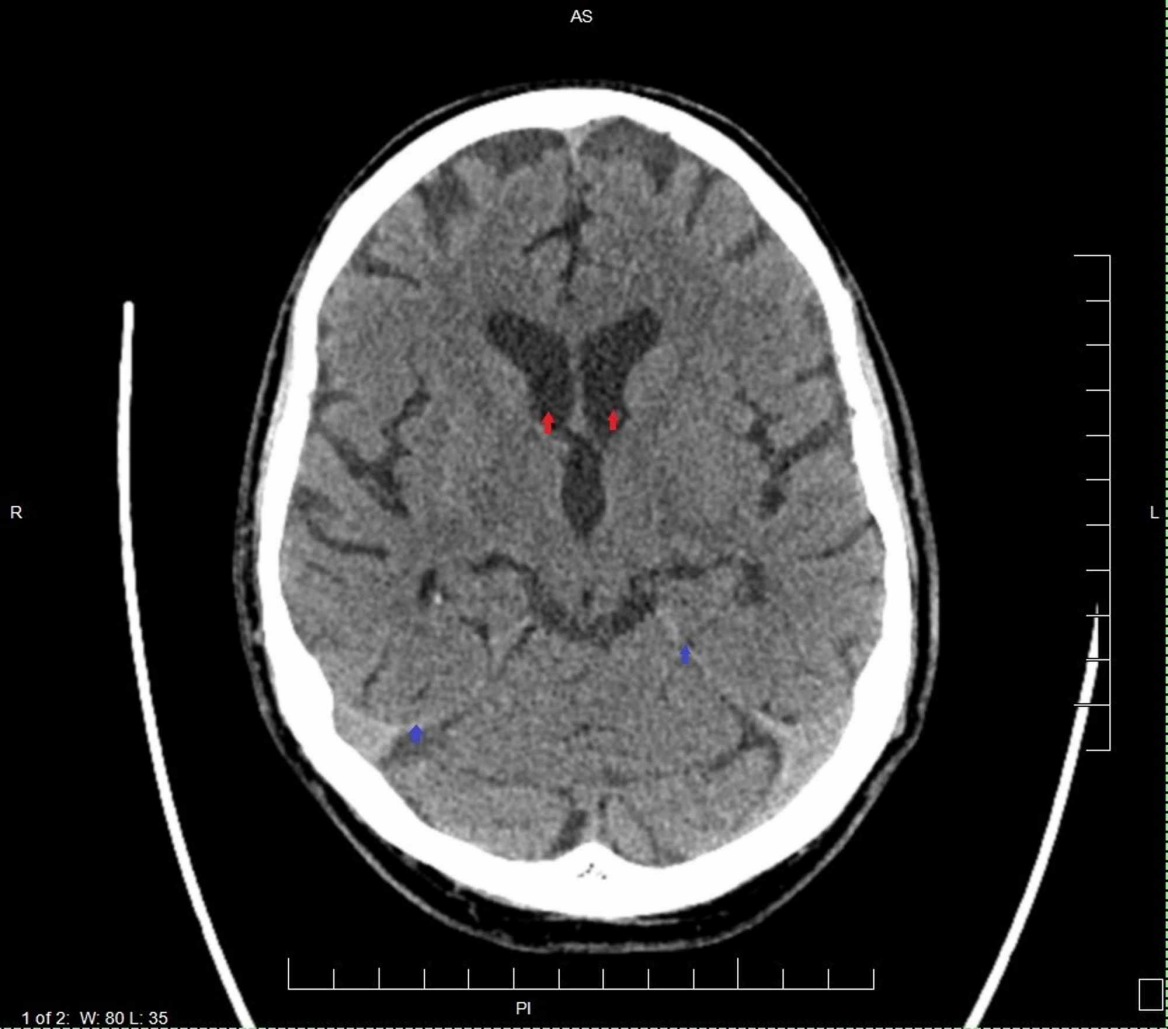
CT scan of the head without contrast. The ventricles and extra-axial spaces are normal (red arrows). No hemorrhage, mass, or mass effect. Normal brain parenchyma. Moderate atherosclerotic calcifications (blue arrows) L: Length; W: Width; R: Right; L: Left; AS: Anterior side; P: Posterior

Urinalysis, urine toxicology, and alcohol screen were negative with thyroid-stimulating hormone result also normal. The patient had a CT scan done, which showed normal ventricles and extra-axial spaces without hemorrhage or mass effect (Figure 1). An electroencephalogram (EEG) revealed generalized background slowing with a mildly slow posterior-dominant rhythm which is consistent with a mild generalized nonspecific cerebral dysfunction. No ictal discharges (seizures) of potential epileptogenicity were seen. MRI findings showed no hemorrhage or acute infarction, no mass lesion, mass-effect, or midline shift. Lateral ventricles were symmetric, without evidence of hydrocephalus. There were normal vascular flow voids and no evidence of abnormal enhancement. No abnormal extra-axial collections and basal cisterns were patent. Visualized paranasal sinuses were also well aerated.

The patient was given olanzapine for his agitation and was subsequently stabilized. On discharge, the patient was deemed not to have decision-making capacity and was ordered to a court-appointed guardian. He was discharged to the care of a senior living facility.

## Discussion

The key feature in GS, though not pathognomonic, is giving approximate answers which our patient exhibits. Our patient often gave approximate answers with little effort, even to simple questions, and responses which varied from day to day. These characteristics are not typical of other causes of cognitive impairment [10]. Approximate answers also known as talking past; passing by; beside the point or near-miss answers are characteristic in some patients [5, 11].

There has been a controversy about whether GS can be attributed to being organic or psychogenic. However, there are reasons to suggest that both causes, on their own, might be the origin of GS.

On one hand, this presentation showed psychogenic characteristics with a major supporting factor being the onset of symptoms right after losing his wife. This is a significant life stressor often associated with GS and other dissociative disorders [12]. With a negative MRI and a CT scan showing moderate atherosclerotic calcifications in a few areas and no acute changes, this alludes to a possible psychogenic cause. The patient also seemed to have selective memory loss of certain topics, like his family members and wife, which is characteristic of a psychological mechanism such as thought blocking [13]. Another aspect supportive to a psychological cause is the rapid onset of the dissociation and approximation of answers to questions. This abrupt change in cognitive functioning, in the absence of acute changes in brain imaging, is typical of a psychological cause and not organic [12]. While most of the symptoms in this patient seem to be explained by the psychogenic nature of the presentation, it is also important to discuss possible and likely organic components playing a role in this pathogenesis. 

With the remote history of traumatic injury which led to hospital admission, there is little doubt that the patient had a probable history of TBI. It is unclear if the exact predisposition of GS is by TBI. Most of the literature has discussed that relative recent brain trauma and acute brain pathology is associated with GS [14]. The assumption that dissociative disorder usually arises from a traumatic event or stressful event [11] might give credence that GS might have been because of this patient’s injury over 40 years ago. Perhaps the history of remote injury to the head is too long ago from his current presentation with no abnormal findings on imaging. The neurobiological cause of GS is still being challenged [11]. Most studies that show organic etiology indicates that the frontal executive function may be accountable for this problem [15-16]. However, this has not been investigated completely as an approximate answer which is an expression of nonaphasic communication disorder is yet to be linked to prefrontal and right hemispheric lesions [11]. Also, dissociative disorders patterns and the psychopathology of GS can be explained by the thalamocorticolimbic disorder model [17]. The EEG findings also showed generalized background slowing with mildly slow posterior-dominant rhythm indicative of cerebral dysfunction; however, the exact role of this finding is unclear in GS.

Ganser syndrome has been described as a dissociative disorder triggered by stressful life events, first described in prisoners, and seen in some immigrants, with a release of glutamate neurotransmitter in the brain [1]. Ouyang et al. suggest that emotional stress can produce glutamate in the corticolimbic tract [1]. Therefore, hyper-glutaminergic transmission in the frontal lobe may cause dissociative symptoms in GS [18]. Kroll et al. postulated that damage to the prefrontal cortex which accounts for confabulation and autobiographical amnesia may result in GS [19]. Therefore, there may be a link between psychogenic and organic causes of GS especially if associated with an emotional stressor with a history of TBI.

## Conclusions

The factors to support a psychogenic cause of this patient’s symptoms are the recent history of major life stressors, the approximation of answers with lack of effort to simple questions, selective memory loss not typical of other forms of cognitive impairment, and the rapid onset of symptoms with no acute anatomical brain changes. However, it is also likely that an organic cause can predispose this patient to developing these symptoms. This case supports the literature that GS is a psychogenic disorder. However, an organic cause from the long-term sequelae of TBI needs further exploration.
